# S-nitrosylation and MSC-mediated body composition

**DOI:** 10.18632/oncotarget.5672

**Published:** 2015-09-15

**Authors:** Yenong Cao, Wayne Balkan, Joshua M. Hare

**Affiliations:** Interdisciplinary Stem Cell Institute, Departments of Molecular and Cellular Pharmacology and Medicine, Miller School of Medicine, University of Miami, Miami, FL, USA

**Keywords:** S-nitrosylation, mesenchymal stem cells, differentiation, PPARgamma

Mesenchymal stem cells (MSCs) are non-hematopoietic stromal cells that can differentiate into multiple mesodermal lineages including adipocytes, osteoblasts, chondrocytes and myocytes. Adult stem cell differentiation is controlled by activation of lineage-specific transcription factors, including peroxisome proliferator-activated receptor γ (PPARγ) and Runx2, two key transcription factors that govern differentiation of MSCs into adipocytes and osteoblasts, respectively. An inverse relationship exists between adipogenesis and osteogenesis. With aging, the differentiation balance in the bone marrow shifts from primarily osteogenesis to adipogenesis, underlying age-related bone loss. Thus, as the population ages and osteoporosis becomes more prevalent, it is crucial to understand the mechanisms that govern the balance between adipogenesis and osteogenesis.

PPARγ, a ligand-activated nuclear receptor required for adipogenesis, is a prominent transcriptional regulator of the bi-lineage differentiation switch of MSCs. PPARγ heterozygous knockout mice have higher bone volume and enhanced osteogenic differentiation of bone marrow cells compared to wild type (WT), indicating that PPARγ exerts inhibitory effects on osteogenesis [1]. Several transcriptional regulators of PPARγ modulate the bi-lineage equilibrium of bone marrow MSCs. However, post-translational regulators of PPARγ-mediated lineage bifurcation remain elusive. Nitric Oxide (NO), which regulates diverse biological functions such as vasodilation, also plays an important role in modulating the balance between adipocyte and osteoblast differentiation. We tested the hypothesis that S-nitrosylation, a crucial component of NO signaling, can modify PPARγ activity and hence the balance between adipogenesis and osteogenesis [2]

## GSNOR and alterations of stem cell lineage and body composition

**Figure 1:**
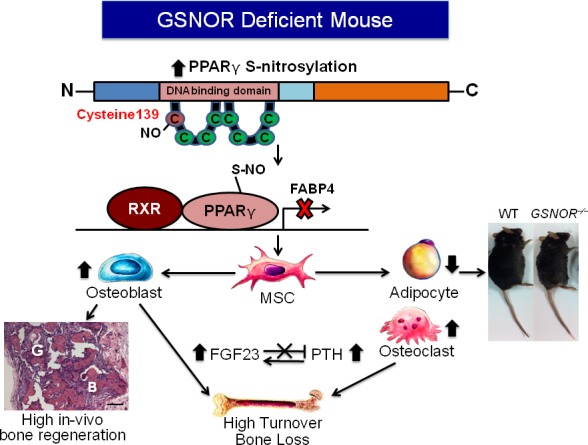
Absence of GSNOR alters the equilibrium of MSC differentiation and PPARγ signaling In GSNOR^−/−^ MSCs, increased S-nitrosylation at Cysteine 139 of PPARγ leads to decreased binding to its downstream target FABP4, contributing to decreased adipogenesis and increased osteogenesis. This phenotype is associated with lower body weight and higher bone regeneration *in vivo*. In addition, GSNOR^−/−^ mice exhibit a concurrent elevation of PTH and FGF23.

S-nitrosylation, a post-translational modification in which NO forms S-nitrosothiols (SNOs) by binding to certain cysteine thiols of protein, is modulated by the denitrosylase, S-nitrosoglutathione reductase (GSNOR). Bone marrow-derived MSCs from mice lacking GSNOR (GSNOR^−/−^ mice) exhibit lower endothelial [3] and adipogenic differentiation and higher osteogenic differentiation [2] than WT cells *in vitro*. Furthermore, in a subcutaneous implantation model, GSNOR^−/−^ MSCs had increased bone regeneration compared to WT MSCs. PPARγ S-nitrosylation at cysteine 139, located within the first zinc finger was enhanced in GSNOR^−/−^ MSCs, thereby reducing PPARγ transcriptional activity and binding to its downstream target FABP4 [2]. Therefore, post-translational modification of PPARγ via S-nitrosylation is a checkpoint regulator of adipogenic-osteogenic lineage bifurcation (Figure 1).

Paradoxically, GSNOR^−/−^ mice are smaller and have lower bone mineral density, despite enhanced bone formation, due to increased bone resorption and higher osteoclast differentiation capacity. These mice also have higher serum parathyroid hormone (PTH) and FGF23 levels and lack the normal inhibitory effects of FGF23 on PTH secretion [2]. The altered hormonal secretion contributes to the high turnover bone loss phenotypes in GSNOR^−/−^ mice and suggests that S-nitrosylation acts as a global regulator of hormonal homeostasis (Figure 1).

In addition to stem cell differentiation, GSNOR also plays a central role in limiting cell proliferation. Increased cell proliferation underlies the resistance of GSNOR^−/−^ mice to myocardial infarction where GSNOR^−/−^ mice exhibit enhanced turnover of cardiomyocytes and cardiac stem cells post-myocardial infarction [4]. These mice also have a high propensity for postnatal hepatocarcinogenesis [5]. This increased cell proliferation suggests that GSNOR and/or protein S-nitrosylation play important roles in regulating cell proliferation.

Our findings also have important implications for the aging population where obesity, bone loss and reduced numbers of functional stem cells are all major concerns. Two-month-old GSNOR^−/−^ mice exhibit osteopenia [2], a disease that is characteristic of aging. Transplantation of young but not old MSCs, to aging mice can slow age-related bone loss and surprisingly prolong the life span of aged mice [6]. Our finding that GSNOR^−/−^ mice have enhanced bone formation and reduced body weight provides therapeutic insights to treat pathological bone loss and weight gain in aging.
